# 1524. SARS-CoV-2 Infection Incidence Following Exposure Assessments for Healthcare Workers

**DOI:** 10.1093/ofid/ofac492.086

**Published:** 2022-12-15

**Authors:** Alon Vaisman, Rob Cairns, Danielle De Graeve, Tamara Dus, Susy S Hota, John Granton

**Affiliations:** University of Toronto, Toronto, Ontario, Canada; University Health Network, Toronto, Ontario, Canada; University Health Network, Toronto, Ontario, Canada; University Health Network, Toronto, Ontario, Canada; University Health Network, Toronto, Ontario, Canada; University Health Network, Toronto, Ontario, Canada

## Abstract

**Background:**

Although healthcare worker (HCW) absenteeism due to COVID-19 exposure represents a significant challenge, there are currently no evidence-based criteria for assessing infection risk based on COVID-19 exposure type. We aimed to identify the incidence of acquiring infection following varying exposures to COVID-19 to guide safe return-to-work policies for staff in healthcare settings.

**Methods:**

We analyzed prospectively collected data at an academic centre with approximately 17 000 active staff between January 1 - April 30, 2022 during a large BA.1 Omicron surge. More than 99% of staff received >2 vaccine doses. All staff self-reporting household, community, and workplace exposure to confirmed cases of COVID-19 submitted attestation to the Occupational Health department detailing the nature of the exposure, the duration, and setting. Staff were required to report all positive test results by rapid antigen or PCR testing.

**Results:**

A total of 3209 staff submitted exposure reports (2493 household, 539 community, and 177 workplace). Of these, 1008 (31.4%) tested positive 2 days prior to or 14 days after the exposure (36% household; 19% community, 7% workplace). In the community exposure group, 19% tested positive due to a discrete exposure of < 4 hours and 21% tested positive with an exposure >4 hours. For household exposures and workplace exposures, these values were 25%/27% and 6%/10%, respectively (Figure 1). The median time to testing positive was 2 days for household exposures and 3 days for community and workplace exposures (Figure 2, Panels A-C). By day 4 post-exposure, more than 80% of positive results were reported (Figure 2, Panel D). Risk of testing positive differed based on baseline symptom status at the time of reporting (Table 1).

Risk of infection during the peri-exposure period (2 days before reported exposure and 14 days after) according to type and duration of exposure. Background rate of infection based on regional incidence of disease due to BA.1 Omicron wave.

SARS-CoV-2 Infection Risk (A - household; B - Community; C - Workplace) after exposure. Time to infection in all groups is shown in panel D.

The risk of infection amongst healthcare workers reporting exposures, according to their symptom status at the time of reporting their exposures.

**Conclusion:**

Our data suggests that the highest risk of acquiring SARS-CoV-2 was via household contacts, regardless of exposure duration, with workplace exposures carrying less risk. Using a cut-off of 4 hours for exposure duration to delineate risk may be of limited value. These data could help workplaces predict infection risk following exposure and guide return-to-work policies that balance the need to staff workplaces, including hospitals, with reducing risk of on-site transmission during periods of increased community transmission (Figure 3).

Risk of Infection in the Context of Background Infection

Background general population infection risk based on regional incidence of disease due to BA.1 Omicron wave.

**Disclosures:**

**All Authors**: No reported disclosures.

